# Sushi Repeat Containing Protein X-linked 2 Is a Downstream Signal of LEM Domain Containing 1 and Acts as a Tumor-Promoting Factor in Oral Squamous Cell Carcinoma

**DOI:** 10.3390/ijms21103655

**Published:** 2020-05-22

**Authors:** Tomonori Sasahira, Miyako Kurihara-Shimomura, Yukiko Nishiguchi, Hiroyuki Shimomura, Tadaaki Kirita

**Affiliations:** 1 Department of Molecular Pathology, Nara Medical University, 840 Shijo-cho, Kashihara, Nara 634-8521, Japan; miyako@naramed-u.ac.jp (M.K.-S.); hurry_archan_iseko@yahoo.co.jp (Y.N.); 2 Department of Oral and Maxillofacial Surgery, Nara Medical University, 840 Shijo-cho, Kashihara, Nara 634-8521, Japan; hiroz@naramed-u.ac.jp (H.S.); tkirita@naramed-u.ac.jp (T.K.)

**Keywords:** oral cancer, sushi repeat containing protein X-linked 2, metastasis, chemosensitivity, angiogenesis, lymphangiogenesis

## Abstract

Because oral squamous cell carcinomas (OSCCs) have a high potential for locoregional invasion and nodal metastasis, early detection and treatment are essential. A LAP2, emerin, MAN1 (LEM) domain containing 1 (LEMD1) is associated with local progression, clinical stage, nodal metastasis, poor prognosis, angiogenesis, and lymphangiogenesis in OSCC. Although LEMD is a cancer-testis antigen, the cancer-related signals related to LEMD1 remain unknown. In this study, we used a microarray analysis of OSCC cells to identify sushi repeat containing protein X-linked 2 (*SRPX2*) as a *LEMD1*-related downstream signal. LEMD1 expression was correlated with lymph node metastasis of OSCC according to the immunohistochemistry analysis. Furthermore, patients expressing SRPX2 had a significantly worse prognosis than those without SRPX2 expression. The concentration of SRPX2 in OSCC was positively correlated with the concentrations of LEMD1, urokinase plasminogen activator receptor (uPAR), and hepatocyte growth factor (HGF). In OSCC cells, SRPX2 secretion levels were elevated by interactions with uPAR and HGF. We also found that SRPX2 promotes endothelial cell proliferation and adhesion between endothelial cells and OSCC cells. These results suggest that SRPX2 might be a useful tumor marker for OSCC.

## 1. Introduction

Oral squamous cell carcinoma (OSCC) is an aggressive cancer with a strong invasiveness and metastatic potential. Globally, approximately 354,864 new OSCC cases and 177,384 deaths were estimated to have occurred in 2018, representing 2% of all cancer cases and 1.9% of all cancer deaths [1]. In the United States, approximately 53,000 new patients are diagnosed with OSCC annually, and 10,860 individuals die of this disease each year [2]. OSCC is also highly prevalent in India, Sri Lanka, and Papua New Guinea, and it is the leading cause of cancer death among men in India and Sri Lanka [1]. Despite advances in diagnostics and therapeutics for OSCC, the five-year survival rates over the last three decades have not improved significantly and have remained lower than 50% [3,4]. Therefore, the molecular biological mechanism of OSCC must be elucidated to improve prognosis through early detection and effective treatment.

LAP2, emerin, MAN1 (LEM) domain containing 1 (LEMD1) is a novel cancer-testis (CT) antigen. Its overexpression has been found in prostate cancer and colon cancer [5,6]. *LEMD1* is a host gene of microRNA-135b (*miR-135b*) and promotes nucleophosmin-anaplastic lymphoma kinase-driven oncogenicity and IL-17–producing immunophenotype in anaplastic large cell lymphoma [7]. LEMD1 also plays a pivotal role in the maintenance of cancer stem cells in colon cancer [8]. Moreover, LEMD1 promotes local progression, poor prognosis, cell cycle progression, cell proliferation, and the inhibition of apoptosis in gastric cancer [9]. We previously reported that LEMD1 expression is strongly correlated with the local progression of tumors, clinical stage, nodal metastasis, and worse outcomes in OSCC patients [10]. Moreover, LEMD1 facilitates cancer cell invasion and promotes the adhesion and transmigration of OSCC cells to vascular and lymphatic endothelial cells. Although LEMD1 may be a novel marker for OSCC, its detailed signaling pathways and further functions in tumors remain unknown.

In this study, we used a cDNA microarray analysis to identify sushi repeat containing protein X-linked 2 (*SRPX2*) as a candidate gene whose expression is upregulated by *LEMD1* in OSCC cells. SRPX2 is a secreted factor that plays important roles in regulating language and cognitive development and synapse formation [11,12]. In previous studies, SRPX2 was shown to act as a tumor-promoting factor in various cancers. SRPX2 overexpression is involved in cell proliferation, invasion, and metastasis in gastric cancer [11,13], colon cancer [14,15], pancreatic cancer [16,17], glioblastoma [18], and osteosarcoma [19], etc. SRPX2 expression is also associated with poor clinical outcomes in several malignancies [13,17,18,20,21]. Recent reports have revealed that the downregulation of *SRPX2* increases chemosensitivity to cisplatin in esophageal cancer [22]. Although cisplatin is usually used to treat OSCC [3], increased drug resistance is a key event leading to cancer progression. SRPX2 may be an indicator of drug resistance in OSCC. Other reports have suggested that SRPX2 is a ligand for urokinase plasminogen activator receptor (uPAR) and that it regulates endothelial cell migration and tube formation in endothelial remodeling during angiogenesis [23,24]. SRPX2 can bind to hepatocyte growth factor (HGF) and promotes tumor angiogenesis [11]. Furthermore, HGF is a lymphangiogenic mediator in cancer [25]. Therefore, the synergy between SRPX2 and LEMD1 may promote angiogenesis and lymphangiogenesis via uPAR and/or HGF in OSCC. However, the function and role of SRPX2 in OSCC remains unknown. 

In the present study, we examined the expression and roles, including the acquisition of drug resistance and angiogenesis, of SRPX2 in OSCC.

## 2. Results

### 2.1. Identification of LEMD1-Related Upregulated or Downregulated Genes in OSCC Cells via a Microarray Analysis

**Figure 1:**
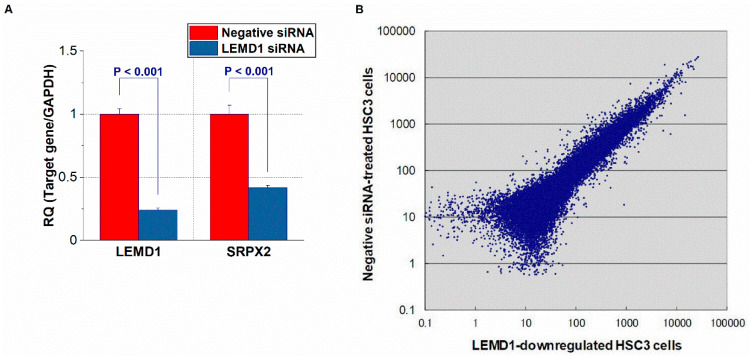
Microarray analysis of HSC3 cells with suppressed *LEM domain containig 1* (*LEMD1*) expression. (**A**) Expression of LEMD1 in control HSC3 cells and HSC3 cells with down regulation of *LEMD1*. (**B**) Scatter plot of gene variation in HSC3 cells and *LEMD1*-downregulated HSC3 cells in the microarray analysis. *p* value < 0.05 was regarded ad statistically significant.

To identify genes whose expression levels are modulated by *LEM domain containing 1 (LEMD1)*, we compared the gene expression profiles of control cells and OSCC cells with reduced *LEMD1* expression using a cDNA microarray analysis (Figure 1A). Data with a low reliability were omitted. The expression levels of many genes were altered by the *LEMD1* knockdown treatment compared with the control OSCC cells (Figure 1B). The ten genes with the most significant changes in upregulation or downregulation in OSCC cells treated with *LEMD1* knockdown relative to the control cells are listed in Table 1. Among these genes, we focused on *SRPX2* because it is one of the genes whose expression levels were greatly reduced in *LEMD1*-downregulated OSCC cells. We confirmed that *LEMD1* knockdown reduce the expression levels of LEMD1 and SRPX2 (Figure 1A). Although this gene is frequently overexpressed in several malignancies [11,13,14,15,16,17,18,19,20,21,24], its expression in OSCC has not been reported. We performed further experiments to investigate the function of SRPX2 in OSCC.

### 2.2. Expression and Prognosis of SRPX2 in OSCC Specimens by Immunohistochemistry 

**Figure 2:**
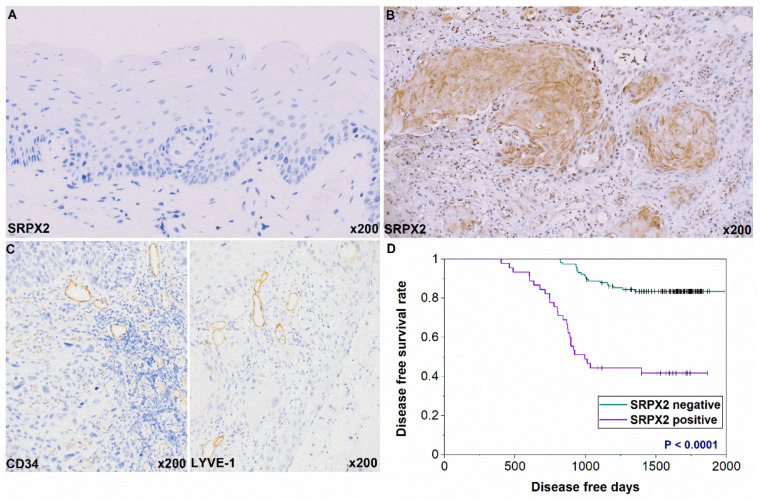
Expression of sushi repeat containing X-linked 2 SRPX2 in oral squamous cell carcinoma (OSCC) patients. (**A**) Weak and/or no SRPX2 expression was detected in normal oral mucosa. (**B**) SRPX2 expression was observed in the cytoplasm in OSCC. (**C**) CD34-positive blood vessels and LYVE-1-positive lymph vessels in OSCC. (**D**) Disease-free survival curve of cases with and without SRPX2 expression. Original magnification was 200×. HE, hematoxylin and eosin.

We next used immunohistochemistry to examine the expression of SRPX2 in 161 OSCC cases. SRPX2 was not present or scarcely expressed in non-neoplastic oral mucosa (Figure 2A). SRPX2 immunoreactivity was observed in 45 of the 161 OSCC cases (28%) and was predominant in the cytoplasm of OSCC cells (Figure 2B). Cytoplasmic expression of SRPX2 is found in other cancers [13,17,19,21], and our results are consistent with those reports. The correlation of SRPX2 with clinicopathological indexes is summarized in Table 2. SRPX2 immunostaining was significantly correlated with nodal metastasis (P = 0.028) in OSCC. Only 27 of the 118 patients (22.9%) without nodal involvement were SRPX2-positive, whereas 18 of 43 patients (41.9%) with nodal metastasis were SRPX2-positive. Further, significant relationships were found between SRPX2 expression and microvessel density (MVD) (P = 0.0111) and lymphovessel density (LVD) (P = 0.0129) (Figure 2C). We also verified that uPAR and HGF expression were strongly related to MVD and/or LVD in the OSCC specimens (submitting). There was no significant association between SRPX2 expression and other clinicopathological factors. 

During the follow-up period, local or nodal recurrence occurred in 45 of the 161 patients. Of the 45 patients with recurrence, 26 (57.8%) displayed SRPX2 expression, whereas only 19 (16.4%) of 116 patients without recurrence were SRPX2-positive (Table 2). We next determined the association between SRPX2 expression and disease-free survival (DFS) in patients with OSCC using the Kaplan–Meier method. The DFS curves showed significantly worse outcomes in cases with SRPX2 expression than in patients who were SRPX2-negative (*p* < 0.0001) (Figure 2D). However, the overall survival rates were not associated with SRPX2 expression levels in OSCC (data not shown).

### 2.3. Gene Expression and Concentration of SRPX2 in OSCC Specimens

**Figure 3:**
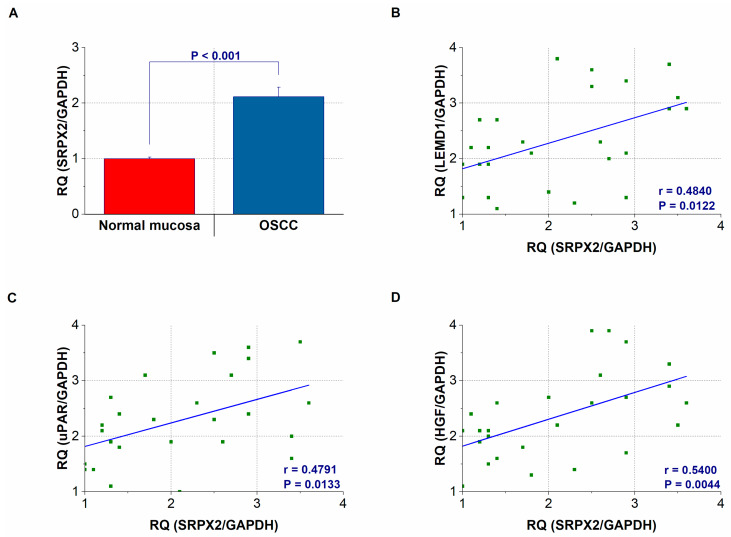
Gene expression and secretion of sushi repeat containing X-linked 2 (SRPX2). (**A**) *SRPX2* expression levels in oral squmous cell carcinoma (OSCC) and non-cancerous oral mucosa. (**B**–**D**) The expression levels of SRPX2 are correlated with those of LEMD1 (**B**), urokinase plasminogen activator receptor (uPAR) (**C**), and hepatocyte growth factor (HGF) (**D**) in OSCC tissues. Error bar, standard deviation (SD). RQ: relative quantification.

We then compared the SRPX2 and LEMD1 expressions in cancerous and surrounding non-cancerous mucosa of 26 OSCC patients. As shown in Figure 3A, the expression levels of SRPX2 were elevated in tissues with OSCC compared with its expression in non-neoplastic mucosal tissues (*p* < 0.001). Additionally, the expression levels of LEMD1 and SRPX2 were strongly correlated in the OSCC tissues (Figure 3B) (*p* = 0.0122). Since SRPX2 is a receptor and binding partner of HGF and uPAR, respectively [11,23,24], we next compared the expression levels of uPAR and HGF. The expression levels of SRPX2 were also strongly correlated with those of uPAR (Figure 3C) (*p* = 0.0133) and HGF (Figure 3D) (*p* = 0.0044) in OSCC tissues. 

### 2.4. Expression of SRPX2 and the Effects of uPAR and HGF on the Secretion of SRPX2 in OSCC Cells

**Figure 4:**
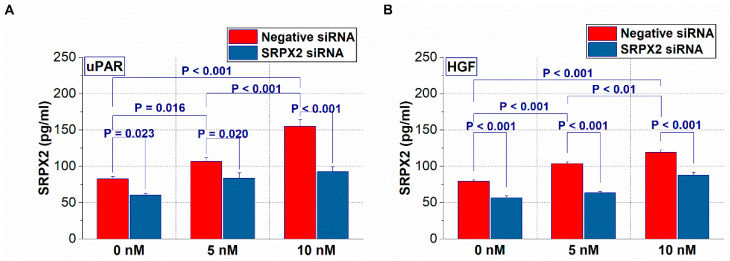
The changes in sushi repeat containing protein X-linked 2 (SRPX2) secretion levels by urokinase plasminogen activator receptor (uPAR) (**A**) and hepatocyte growth factor (HGF) (**B**) recombinant proteins in HSC3 cells. Error bar, standard deviation (SD).

Subsequently, we performed a cell-based analysis. Since SRPX2 is a secretory factor [11,12], we next investigated the secretion status of SRPX2 in the presence of uPAR or HGF in OSCC cells by enzyme-linked immunosorbent assay (ELISA). As shown in Figure 4, OSCC cells with recombinant uPAR or HGF treatment increased the secretion of SRPX2 into the culture supernatant, whereas its effect was suppressed by neutralization treatment with SRPX2 antibody.

### 2.5. Effect of SRPX2 on Angiogenesis and Lymphangiogenesis of OSCC Cells

**Figure 5:**
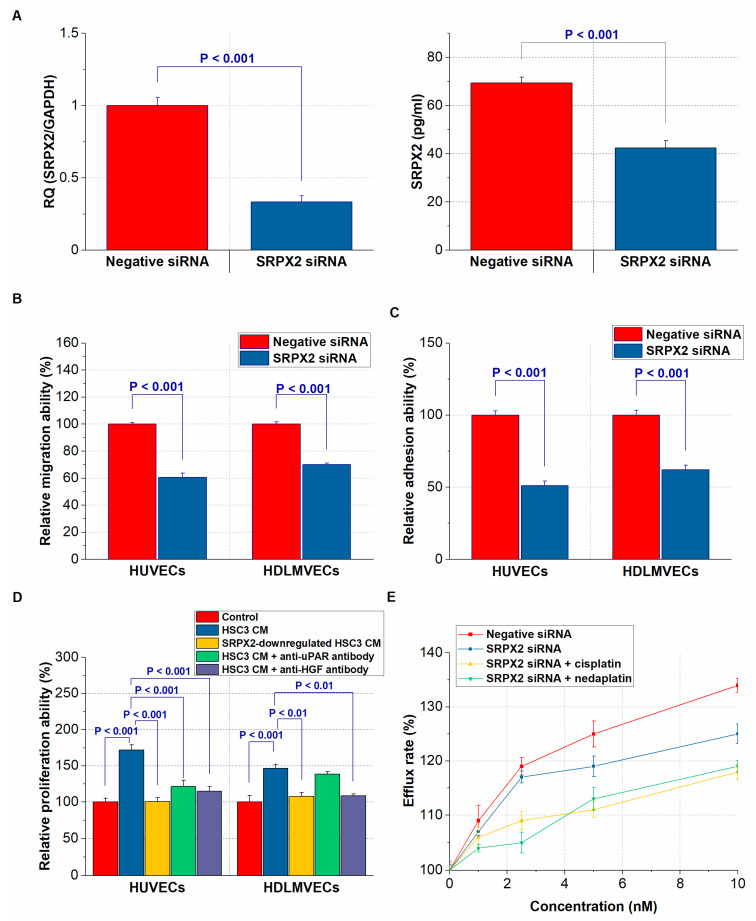
In vitro functional analysis of *sushi repeat containing X-linked 2 (SRPX2)* on endothelial cells and chemosensitivity. (**A**) The expression and secretion change of SRPX2 by *SRPX2*-knockdown treatment in HSC3 cells supernatant. (**B**) The regulation of migration of human umbilical vain endothelial cells (HUVECs) and human dermal lymphatic microvascular endothelial cells (HDLMVECs) by *SRPX2*. (**C**) The modulation of adhesion between endothelial cells and HSC3 cells by *SRPX2*. (**D**) The conditioned medium (CM) of SRPX2-producing HSC3 cells promotes the proliferation of endothelial cells, whereas their effects are offset by uPAR and HGF antibodies. (**E**) The influence of *SRPX2* on the resistance of HSC3 cells to platinum agents.

Next, we investigated the effect of *SRPX2* knockdown on the interaction between HSC3 cells and endothelial cells (Figure 5A). The downregulation of *SRPX2* in HSC3 cells caused a decrease in transmigration ability of human umbilical vein endothelial cells (HUVECs) and human dermal lymphatic microvascular endothelial cells (HDLMVECs) (Figure 5B). Furthermore, adhesion ability to HUVECs and HDLMVECs was also suppressed in HSC3 cells with decreased expression of *SRPX2* (Figure 5C). We next confirmed the influence of the CM of SRPX2-producing HSC3 cells on cell growth in endothelial cells. As shown in Figure 5D, the cell growth of endothelial cells was significantly enhanced by the addition of CM derived from control HSC3 cells, while uPAR antibody treatment was suppressed the proliferation ability of HUVECs. The growth ability of HUVECs and HDLMVECs was also suppressed by HGF antibody treatment. We speculated that this was because HGF was involved in lymphangiogenesis as well as angiogenesis [11,25]. Furthermore, the treatment of endothelial cells with the CM from *SRPX2*-downregulated HSC3 cells resulted in attenuation of cell proliferation. 

### 2.6. Effect of SRPX2 on the Drug Resistance of OSCC Cells

Since SRPX2 is related to cisplatin resistance in esophageal cancer cells [22], we finally verified the effects of SRPX2 on OSCC cell tolerance to platinum anticancer drugs. We used a multidrug-resistance assay kit that can measure the efflux of a fluorescent dye that binds to cell surface ATP-binding cassette (ABC) transporters. We have already confirmed that OSCC cells overexpress ABC transporters [3]. *SRPX2* knockdown restored sensitivity to nedaplatin and cisplatin in OSCC cells (Figure 5D). Additionally, co-treatment with *SRPX2* siRNA and platinum anticancer drugs reduced drug resistance in a dose-dependent manner. However, we did not identify any associations (data not shown). In summary, the results suggest that SRPX2 may act as a tumor-promoting factor for OSCC by acquiring angiogenic and lymphangiogenic abilities and creating anticancer drug resistance.

## 3. Discussion

LEMD1 is a CT antigen that is overexpressed in several malignancies [5,6,7,8,9]. We previously reported that LEMD1 is closely involved in tumor progression, nodal metastasis, and worse OSCC outcomes by acquiring invasiveness and angiogenic and lymphangiogenic potential [10]. Because CT antigens are useful targets for cancer vaccination and immunotherapy through the activation of cytotoxic T lymphocytes [26], *LEMD1* may be a useful candidate for molecular targeted therapy for OSCC. However, little is known about *LEMD1* downstream signaling in cancer. In our present study, we used a cDNA microarray to perform gene expression profiling, and we identified *SRPX2* as a novel gene related to *LEMD1*. We also showed that SRPX2 contributes to reinforced drug resistance to platinum agents, angiogenesis, and lymphangiogenesis in OSCC cells. Cisplatin induces cancer cell death by inhibiting DNA replication, and nedaplatin is a platinum drug with less nephrotoxicity [3,22]. Combination therapy centered on cisplatin is often given as the first-line chemotherapy for OSCC, whereas chemoresistance remains a major problem in OSCC treatment [3]. Moreover, cetuximab, an anti-epidermal growth factor receptor (EGFR)-specific chimeric monoclonal antibody, and nivolumab, an anti-programmed cell death-1 receptor (PD-1)-specific monoclonal antibody, are the only molecularly targeted drugs used for OSCC, and their therapeutic effects remain controversial [3,27]. Our study has demonstrated that the LEMD1-SRPX2 system is associated with resistance to cisplatin and nedaplatin in OSCC cells. However, further in vitro and animal studies are necessary to clarify the link between SRPX2 and other chemotherapeutic drugs, radiation therapy, etc.

There was a significant correlation between SRPX2 expression and lymph node metastasis in the immunohistochemical analysis of OSCC patients. Additionally, the DFS of SRPX2-positive cases determined via immunostaining was significantly worse than that of SRPX2-negative cases. In the gene expression analysis, *SRPX2* was more highly expressed in cancer tissues than in normal oral mucosa, and in cancer tissues, *SRPX2* expression levels were significantly correlated with those of *LEMD1*. Our present results are generally consistent with those of previous reports of other cancers and SRPX2. SRPX2 acts as a tumor-promoting factor that promotes proliferation, invasion, and metastasis in various cancers [11,13,14,15,16,17,18,19]. Further, *SRPX2* expression is implicated in poor prognoses of cancer of the stomach [13], pancreas [17], liver [20], and prostate [21], and in glioblastoma [18] and osteosarcoma [19]. The following ten key alterations are fundamental to malignant transformation and cancer progression: self-sufficiency in proliferative signals, insensitivity to growth-inhibitory signals, the avoidance of destruction of the immune system, the ability to invade and metastasize, tumor-promoting inflammation, limitless replicative capacity, sustained angiogenesis, genetic instability and mutation, resistance to cell death, and deregulated cellular metabolism [28]. Several studies have been conducted to date regarding the above ten changes in cancer. However, OSCC-specific diagnostic and therapeutic targets require further elucidation. A differential comprehensive gene expression analysis will be beneficial for discovering novel tumor markers. Further large-scale studies using clinical samples (tissue sections, frozen material, blood, saliva, etc.) will be essential for the usefulness of SRPX2 as a tumor marker for OSCC.

SRPX2 is a secreted protein that contains three sushi motifs. It is involved in protein–protein or protein–carbohydrate interactions. It also contains one hyaline domain, which appears to be related to cellular adhesion [11,13,23]. SRPX2 binds to uPAR via a ligand/receptor interaction, and SRPX2 mutations have been shown to lead to an increased SRPX2/uPAR binding affinity [11]. More interestingly, SRPX2 can promote the proliferation ability of endothelial cells by binding to HGF in a dose-dependent manner [11]. The expression levels of *SRPX2* in our OSCC samples were well correlated with those of not only *LEMD1* but also *uPAR* and *HGF*. The secretion levels of SRPX2 in the ELISA of our OSCC samples were well correlated with those of not only LEMD1 but also uPAR and HGF. Further, SRPX2 secretion levels were increased in OSCC cells containing recombinant proteins of uPAR and HGF in the culture medium. Our results suggest that the interaction of SRPX2 with uPAR and HGF may increase the secretory levels of SRPX2 via an autocrine loop in OSCC cells. SRPX2 expression is regulated by *miR-149* [14] and *miR-192/215* [15], but the mechanism of SRPX2 expression regulation and activation in cancer is not well understood. Interestingly, *miR-149* has been shown to be involved in tumor progression by regulating the interleukin-6 (IL-6)/signal transducer and activator of transcription 3 (STAT3) pathway in esophageal [29] and gastric cancer [30]. In addition, IL-6 is a useful salivary biomarker in OSCC and its expression in cancer tissues has also been shown to be involved in tumor progression [31]. LEMD1-SRPX2-IL-6 signaling may be an important pathway in the development and growth of OSCC. Further research is being conducted to clarify the activation mechanism of SRPX2 in malignancies.

Previous reports have demonstrated that the CM of SRPX2-producing cancer cells strikingly intensifies the cell proliferation of vascular endothelial cells [11,24]. In this study, CM derived from HSC cells markedly enhanced the proliferative capacity of HUVECs and HDLMVECs, whereas their effects were offset by neutralization with uPAR and/or HGF antibodies. Moreover, SRPX2 was revealed to regulate the growth of vascular and lymphatic endothelial cells and adhesion between HSC3 cells and endothelial cells. Since the interaction between SRPX2 and uPAR is facilitated by angiogenesis [23,24] and because HGF is involved in both lymphangiogenesis and angiogenesis [11,25], our results almost fall in line with those reports. Angiogenesis, the growth of new blood vessels, and lymphangiogenesis, the development of new lymphatic vessels, are pivotal events for tumor progression and metastasis [32]. The vascular endothelial growth factor (VEGF) family has been shown to play essential roles in angiogenesis and lymphangiogenesis, and we have demonstrated that strong expression levels of VEGF-A, -C, and -D are markedly related to tumor progression, nodal metastasis, and poor prognosis [32]. However, the mechanisms and roles of angiogenesis and lymphangiogenesis are extremely complex, and their significance in OSCC remains unclear. Our results suggest that SRPX2 promotes vascular and lymphatic endothelial cell migration, the adhesion of HUVECs and HDLMVECs to cancer cells, and the elevation of MVD and LVD in OSCC. Compared with normal blood vessels, tumoral irregular blood vessels lack pericytes and are attenuated by the delivery of anticancer drug [32,33]. A recent report has suggested that antiangiogenic gene therapy is useful for the prevention and early treatment of malignancies [34]. LEMD1-SRPX2 signaling might be a useful target for anti-angiogenic and lymphangiogenic therapy for OSCC; thus, further studies on this topic are desired. 

In summary, the main results of this study are as follows: (1) *SRPX2* is a novel *LEMD1*-related gene in OSCC, (2) SRPX2 expression is associated with nodal metastasis and the prognosis of OSCC patients, (3) the secretion of SRPX2 from OSCC cells is enhanced by uPAR and HGF, and (4) SRPX2 induces resistance to platinum-based anticancer agents and vascular and lymphangiogenesis in OSCC. These results indicate that SRPX2 can serve as a molecular target for OSCC diagnosis and treatment. However, it is also true that this study is still developing, and there are limits to its conclusions. A detailed analysis on further molecular biological functions and roles of SRPX2 will be performed in future studies.

## 4. Materials and Methods 

### 4.1. Cell Culture

The human OSCC cell lines HSC3 were obtained from the Japanese Collection of Research Bioresources (JCRB) Cell Bank, Osaka, Japan. The cells were authenticated by the JCRB via a short tandem repeat analysis. The cells were maintained in Dulbecco’s modified Eagle’s medium (Wako Pure Chemical, Osaka, Japan) supplemented with 10% fetal bovine serum (Nichirei Biosciences, Tokyo, Japan) under conditions of 5% CO_2_ in air at 37 °C.

Primary HUVECs and primary HDLMVECs were purchased from Cell Applications (San Diego, CA, USA). The HUVECs were cultured in endothelial cell media (Cell Applications), and the HDLMVECs were cultured in microvascular endothelial cell media (Cell Applications), both with 5% CO_2_ at 37 °C. 

### 4.2. Microarray Analysis

We have previously confirmed that OSCC cells express *LEMD1* and that knockdown treatment reduces its expression level [10]. For the microarray analysis, we used HSC3 cells without treatment and with suppressed expression of *LEMD1*. Total RNA was isolated using a RNeasy Mini kit (Qiagen, Venlo, Limburg, The Netherlands) according to the manufacturer’s instructions. Preparations were quantified, and their purity was determined by standard spectrophotometric methods. A CodeLink™ Human Whole Genome Bioarray (Applied Microarrays, Tempe, AZ, USA) containing 54,841 probes was used to compare transcriptional profiles between control HSC3 cells and LEMD1 downregulated HSC3 cells. The data analysis was performed with Microarray Data Analysis Tool Ver3.2 (Filgen, Nagoya, Japan). Quantile normalization was performed. The microarray data used in this study have been deposited in the national center for biotechnology information (NCBI) Gene Expression Omnibus (GEO) database (http://www.ncbi.nlm.nih.gov/geo/) and can be accessed through GEO Series accession number GSE80347.

### 4.3. Tumor Specimens

Formalin-fixed, paraffin-embedded samples from 161 primary OSCC cases (89 males and 72 females, mean age 65.6 years, range 42–88 years) were evaluated via immunohistochemistry. Fresh-frozen samples from 26 OSCC cases and corresponding non-neoplastic mucosa samples were utilized for the quantitative reverse transcription PCR (qRT-PCR) analysis. All specimens were preoperatively untreated cases that were chosen at random from the Nara Medical University Hospital, Kashihara, Nara, Japan. Tumor stage and OSCC histological grade were determined according to the UICC TNM classification system, 8th edition [35], and WHO criteria [36], respectively. Medical records were obtained from the patient database managed by the hospital. Written informed consent for using the tissue samples was obtained from the patients. All experiments with human samples were performed according to the Declaration of Helsinki and were approved by the Ethical Committee of Nara Medical University (Approval number. 719).

### 4.4. Immunohistochemistry

Consecutive 3-μm sections were cut from each block and subjected to immunohistochemical staining. An immunoperoxidase technique was applied following antigen retrieval with microwave treatment (95 °C) in citrate buffer (pH 6.0) for 45 min. The sections were pretreated with 3% H_2_O_2_-methanol to block endogenous peroxidase activity at room temperature, and the specimens were incubated in 10% skim milk solution (Morinaga Milk, Tokyo, Japan) for 20 min at room temperature to avoid false-positive antibody reactions. Anti-SRPX2 (LifeSpan BioSciences, Seattle, WA, USA; dilution at 1:100), anti-CD34 (Abcam, Cambridge, UK; dilution at 1:150), and anti-LYVE-1 antibodies (Abcam; dilution at 1:100) were used as the primary antibodies. After incubating overnight at 4 °C, the sections were incubated with the secondary antibody, peroxidase-conjugated anti-rabbit antibodies (Medical & Biological Laboratories, Nagoya, Japan; dilution at 1:200), at room temperature for 30 min. The specimens were color-developed with diaminobenzidine (DAB) solution (Dako, Carpinteria, CA, USA) and counterstained with Meyer’s hematoxylin (Sakura Finetek, Tokyo, Japan). 

The immunoreactivity of SRPX2 was classified according to Allred’s score (AS) [29] and divided into four grades: Grade 0, AS = 0; Grade 1, AS = 2–4; Grade 2, AS = 5–6; and Grade 3, AS 7–8. Cases classified as Grade 2 and 3 were considered immunologically positive [10,37]. The MVD and LVD were measured on anti-CD34 and anti-LYVE-1 antibody immunopositive specimens, respectively. To quantify MVD or LVD, five maximum vessel density fields were selected from around the tumor cells (the “hot spot”) and examined under microscopy at 200-fold magnification; the results were averaged [32].

### 4.5. RNA Extraction and Quantitative Reverse-Transcription Polymerase Chain Reaction

Total RNA was extracted using an RNeasy Mini kit (Qiagen), and 1 μg of total RNA and the ReverTra Ace qPCR RT Kit (Toyobo, Osaka, Japan) were used to synthesize the cDNA. qRT-PCR was performed on a StepOnePlus Real-Time PCR System (Applied Biosystems; Thermo Fisher Scientific) with TaqMan Fast Universal PCR Master Mix (Applied Biosystems; Thermo Fisher Scientific), and the results were analyzed using the relative standard curve quantification method. Glyceraldehyde-3-phosphate dehydrogenase (*GAPDH*) mRNA was used as the internal control. The TaqMan Gene Expression Assays for *SRPX2* (identification number: Hs00997585_m1), *LEMD1* (identification number: Hs01077215_m1), and *GAPDH* (identification number: Hs03929097_g1) were purchased from Applied Biosystems (Thermo Fisher Scientific). 

### 4.6. ELISA for SRPX2, uPAR, and HGF

A small piece (approximately 10 mm^3^) of homogenized tumor tissues and cell culture medium was collected and centrifuged at 4 °C for 10 min. Proteins in the supernatant were then extracted using M-PER Mammalian Protein Extraction Reagent. An ELISA was used to measure the protein concentrations of SRPX2 (LifeSpan BioSciences), uPAR (Abcam), and HGF (Proteintech Rosemont, IL, USA). The assays were performed according to the manufacturers’ instructions and in triplicate. The presented data are the means of three independent experiments.

### 4.7. Small Interfering RNA

To suppress endogenous gene expression, the cells were treated with small interfering RNA (siRNA). Silencer Select RNAi for SRPX2 (identification number: s26078) and LEMD1 (identification number: s41117) was purchased from Ambion (Thermo Fisher Scientific), and AllStars Negative Control siRNA (Qiagen) was used as a control. The cells were transfected with 10 nmol of siRNA using Lipofectamine 2000 (Invitrogen; Thermo Fisher Scientific) according to the manufacturer’s recommendations.

### 4.8. Treatment with Recombinant Protein and SRPX2-Conditioned Medium

The HSC3 cells were treated with 0, 5, and 10 nM recombinant human uPAR (Abnova, Taipei, Taiwan) and HGF (Abnova) for 48 h at 37 °C, and an ELISA was performed. To generate CM, negative siRNA or *SRPX2* siRNA-treated HSC3 cells were incubated at 37 °C. The medium of the sub-confluent HSC3 cells was changed to Opti-MEM reduced serum medium (Thermo Fisher Scientific, Waltham, MA, USA), and the CM was collected after further culturing for 24 h [24,37].

### 4.9. Cell Proliferation of Endothelial Cells and Assays of Interactions between OSCCs and Endothelial Cells

Endothelial cells were cultured with only endothelial growth media, microvascular endothelial growth media, or each CM and seeded at a density of 2000 cells per well in 96-well tissue culture plates and incubated for 48 h at 37 °C. Anti-uPAR (Santa Cruz Biotechnology, Santa Cruz, CA, USA) and anti-HGF antibodies (Santa Cruz Biotechnology) were added at a concentration of 2 μL/mL for 2 h to endothelial cells cultured in CM and treated with negative siRNA. A cell growth assay was performed using Cell Counting Kit-8 (Dojindo Laboratories, Kumamoto, Japan) [37]. The interactions of HSC3 cells and endothelial cells were tested using a CytoSelect Tumor-Endothelium Adhesion Assay (Cell Biolabs, San Diego, CA, USA) and CytoSelect Tumor Transendothelial Migration Assay systems (Cell Biolabs), as described in our previous reports [3,10]. Briefly, a suspension of fluorescently labeled OSCC cells was cultured with a monolayer of endothelial cells and lysed in lysis buffer. Absorbance at 450 nm (to measure cell growth) and 480/520 nm (to measure adherent or migrating cells) was determined using a Multiskan GO Microplate Spectrophotometer (Thermo Fischer Scientific). 

### 4.10. Anticancer Resistance Assays 

HSC3 cells were treated with 0, 1, 2.5, 5, and 10 μM cisplatin (Wako Pure Chemical) or nedaplatin (Wako Pure Chemical). After 24 h, drug resistance was monitored using a MarkerGene Multiple Drug Resistance Microtiterplate Assay Kit (Marker Gene Technologies, Eugene, OR, USA). Drug resistance was measured with a SpectraMax M2 multi-detection microplate reader (Molecular Devices, Sunnyvale, CA, USA) at an emission wavelength of 504 nm and an excitation wavelength of 538 nm.

### 4.11. Statistical Analysis

The statistical analyses were performed using Fisher’s exact test, the Student’s t-test, Welch’s t-test, and the Mann–Whitney U-test. The DFS was analyzed using the Kaplan–Meier method and compared among groups using the log-rank test. All statistical analyses were conducted using JMP13 (SAS Institute, Cary, NC, USA), and a P value < 0.05 was considered statistically significant.

## Figures and Tables

**Table 1:** The Ten Most Unregulated or Downregulated Genes Except *LEMD1* in *LEMD1* Knockdown OSCC Cells Compared to Control Cells.

Downregulated		Upregulated	
Gene	Fold (*LEMD1* Knockdown/Control)	Gene	Fold (*LEMD1* Knockdown/Control)
*CEACAM6*	0.054	*MRPL2*	12.481
*ESM1*	0.066	*GSX2*	10.285
*PHLDA1*	0.101	*KRT5*	9.615
*BIRC3*	0.113	*BIRC6*	8.474
*CALB2*	0.115	*GSTA4*	8.446
*DHRS9*	0.121	*FOS*	7.613
*KPNA7*	0.130	*AKR1B10*	7.591
*SOX9*	0.135	*BOLA3*	7.575
*SERPINE2*	0.140	*AHSG*	7.131
*SRPX2*	0.154	*DST*	6.435

**Table 2:** Relationship between SRPX2 Expression and Clinicopathological Parameters.

	SRPX2 Expression		
Parameters	− (%)	+ (%)	*p* Value *
Gender			
Male	61 (68.5)	28 (31.5)	
Female	55 (76.4)	17 (23.6)	0.2938
Age			
<65	54 (75)	18 (25)	
>65	62 (69.7)	27 (30.3)	0.4844
Site			
Tongue	60 (66.7)	30 (33.3)	
Gingiva	42 (80.8)	10 (19.2)	
Buccal mucosa	7 (70)	3 (30)	
Other	7 (77.8)	2 (22.2)	0.3305
Histology			
Well	57 (75)	19 (25)	
Moderately, Poorly	59 (69.4)	26 (30.6)	0.4839
T classification			
T1-T3	81 (74.3)	28 (25.7)	
T4	35 (67.3)	17 (32.7)	0.3554
Clinical stage			
I-II	79 (75.2)	26 (24.8)	
IV	37 (66.1)	19 (33.9)	0.2688
Nodal metastasis			
Negative	91 (77.1)	27 (22.9)	
Positive	25 (58.1)	18 (41.9)	0.0280
MVD	25.128 ± 14.741	33.109 ± 23.841	0.0111
LVD	20.245 ± 13.894	27.136 ± 19.583	0.0129

The relationship between expression of sushi repeat containing protein X linked-2 (SRPX2) and each factor was calculated by Fisher’s exact test or chi-square test. The differences of the microvessel density (MVD) and lymphovessel density (LVD) among groups were analyzed by unpaired parametric *t*-test. MVD and LVD were Means ± S.D. (standard deviation), each S.D. was less than 10% in all cases. T classification and clinical stage were classified according to the TNM classification.* *p* value < 0.05 was regarded as statistically significant.

## Abbreviations

OSCC	oral squamous cell carcinoma
LEMD 1	LEM domain containing 1
SRPX2	sushi repeat containing protein X-linked 2
uPAR	urokinase plasminogen activator receptor
HGF	hepatocyte growth factor
MVD	microvessel density
LVD	lymphovessel density
